# *trans*-Tetra­aqua­bis­[1,3-bis­(4-pyrid­yl)propane-κ*N*]cobalt(II) biphenyl-4,4′-disulfonate monohydrate

**DOI:** 10.1107/S1600536811015819

**Published:** 2011-04-29

**Authors:** Guang-Xiang Liu, Xu-Yong Xu

**Affiliations:** a School of Chemistry and Chemical Engineering, Anqing Normal University, Anqing 246003, People’s Republic of China

## Abstract

In the title compound, [Co(C_13_H_14_N_2_)_2_(H_2_O)_4_](C_12_H_8_O_6_S_2_)·H_2_O, the cation, anion and uncoordinated water mol­ecule have crystallographically imposed twofold symmetry. The cobalt(II) atom exhibits a slightly distorted octa­hedral coordination geometry provided by two N atoms from two 1,3-bis­(4-pyrid­yl)propane ligands and the O atoms from four water mol­ecules. The dihedral angle between the pyridine rings in the ligand is 86.14 (11)°, whereas the dihedral angle formed by the symmetry-related benzene rings in the anion is 35.81 (12)°. In the crystal, cations, anions and water mol­ecules are linked into layers parallel to the *ac* plane by O—H⋯O and O—H⋯N hydrogen-bond inter­actions. The layers are further connected into a three-dimensional network by C—H⋯O hydrogen bonds.

## Related literature

For applications of bipyridine ligands and the 4,4′-biphenyl­disulfonate dianion in coordination chemistry, see: Lu *et al.* (2006 ▶); Ghoshal *et al.* (2003 ▶); Brandys & Puddephatt (2001 ▶); Tong *et al.* (2002 ▶); Wang *et al.* (2005 ▶); Suresh & Bhadbhade (2001 ▶); Mago *et al.* (1997 ▶); Pan *et al.* (2001 ▶); Chen, Cai, Feng & Chen (2002 ▶); Chen, Cai, Liao *et al.* (2002 ▶); Lian, Cai & Chen (2007 ▶); Lian, Cai, Chen & Luo (2007 ▶); Liu *et al.* (2010 ▶).
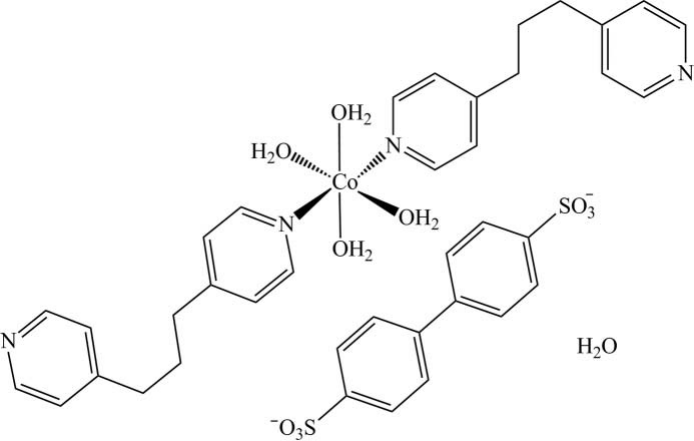


## Experimental

### 

#### Crystal data

[Co(C_13_H_14_N_2_)_2_(H_2_O)_4_](C_12_H_8_O_6_S_2_)·H_2_O
*M* *_r_* = 857.84
Monoclinic, 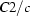

*a* = 15.555 (3) Å
*b* = 18.983 (3) Å
*c* = 14.725 (3) Å
β = 113.959 (3)°
*V* = 3973.3 (12) Å^3^
*Z* = 4
Mo *K*α radiation
μ = 0.60 mm^−1^
*T* = 293 K
0.28 × 0.24 × 0.22 mm

#### Data collection

Bruker SMART APEX CCD area-detector diffractometer
Absorption correction: multi-scan (*SADABS*; Bruker, 2000 ▶) *T*_min_ = 0.850, *T*_max_ = 0.879
10176 measured reflections
3683 independent reflections
3035 reflections with *I* > 2σ(*I*)
*R*_int_ = 0.031

#### Refinement

*R*[*F* ^2^ > 2σ(*F* ^2^)] = 0.054
*wR*(*F* ^2^) = 0.133
*S* = 1.04
3683 reflections
274 parameters
H atoms treated by a mixture of independent and constrained refinement
Δρ_max_ = 0.53 e Å^−3^
Δρ_min_ = −0.22 e Å^−3^

### 

Data collection: *SMART* (Bruker, 2000 ▶); cell refinement: *SAINT* (Bruker, 2000 ▶); data reduction: *SAINT*; program(s) used to solve structure: *SHELXS97* (Sheldrick, 2008 ▶); program(s) used to refine structure: *SHELXL97* (Sheldrick, 2008 ▶); molecular graphics: *SHELXTL* (Sheldrick, 2008 ▶); software used to prepare material for publication: *SHELXTL*.

## Supplementary Material

Crystal structure: contains datablocks I, global. DOI: 10.1107/S1600536811015819/rz2587sup1.cif

Structure factors: contains datablocks I. DOI: 10.1107/S1600536811015819/rz2587Isup2.hkl

Additional supplementary materials:  crystallographic information; 3D view; checkCIF report

## Figures and Tables

**Table 1:** Hydrogen-bond geometry (Å, °)

*D*—H⋯*A*	*D*—H	H⋯*A*	*D*⋯*A*	*D*—H⋯*A*
O1*W*—H1*WA*⋯O3^i^	0.81 (4)	2.60 (4)	3.008 (4)	113 (3)
O1*W*—H1*WA*⋯O1^i^	0.81 (4)	2.01 (5)	2.812 (4)	169 (4)
O2*W*—H2*WA*⋯N2^ii^	0.86 (5)	1.93 (5)	2.779 (4)	167 (5)
O1*W*—H1*WB*⋯O3^iii^	0.71 (4)	2.01 (5)	2.687 (4)	160 (5)
O2*W*—H2*WB*⋯O2^iii^	0.78 (4)	2.01 (4)	2.795 (4)	179 (4)
O3*W*—H3*W*⋯O1^iv^	0.87 (6)	2.05 (6)	2.924 (4)	174 (7)
C10—H10⋯O2^v^	0.93	2.56	3.360 (4)	144
C16—H16⋯O3*W*^vi^	0.93	2.54	3.311 (5)	141

Symmetry codes: (i) 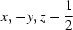; (ii) 
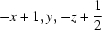
; (iii) 
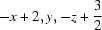
; (iv) 
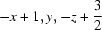
; (v) 
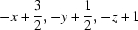
; (vi) 
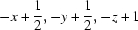
.

**Fig. 1.**
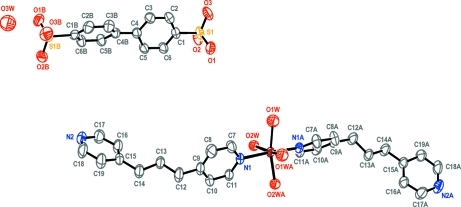
The structure of the title compound, showing 50% probability displacement ellipsoids. Hydrogen atoms are omitted for clarity [symmetry codes: (A) 2-x, y, 0.5-z; (B) 1-x, y, 1.5-z].

**Fig. 2.**
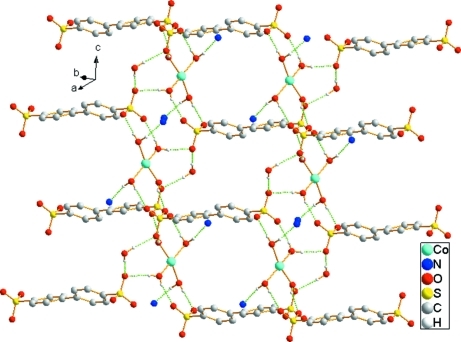
The two-dimensional network formed by hydrogen-bonding interactions (green dotted lines). For clarity, the bpp ligands and hydrogen atoms attached to carbon atoms are omitted.

**Fig. 3.**
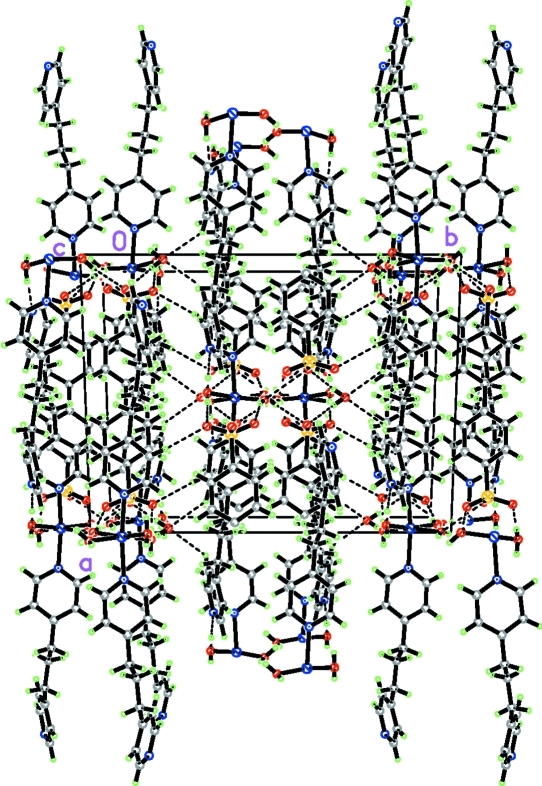
The three-dimensional network of the title complex. Hydrogen bonds are shown as blue dotted lines.

## supplementary crystallographic information

### Comment

Bipyridine ligands with certain spacers between the two terminal coordination groups, for example 4,4-bipyridine (bpy), 1,2-bis(4-pyridyl)ethane (bpe), 1,2-di(4-pyridyl)ethylene (dpe), and 1,3-bi(4-pyridyl)propane (bpp), have been employed to construct novel metal-organic coordination polymers with beautiful aesthetics and useful functional properties. (Lu *et al.*, 2006; Ghoshal *et al.*, 2003; Brandys & Puddephatt, 2001; Tong *et al.*, 2002; Wang *et al.*, 2005; Suresh & Bhadbhade, 2001; Mago *et al.*, 1997; Pan *et al.*, 2001). The 4,4'-biphenyldisulfonate dianion (BPDS^2-^), which possesses six oxygen atoms, has been also employed either as a ligand with multiple binding sites available to construct coordination polymers with varying dimensionalities, or as a counter ion, forming extensive hydrogen-bonding interaction with the water molecules (Chen, Cai, Feng & Chen, 2002; Chen, Cai, Liao & Feng, 2002; Lian, Cai & Chen 2007; Lian, Cai, Chen & Luo 2007; Liu *et al.*, 2010). In the present work, we report a cobalt(II) complex, [Co(C_13_H_14_N_2_)_2_(H_2_O)_4_](C_12_H_8_O_6_S_2_).H_2_O (I), with a two-dimensional H-bonding network structure created by the sulfonate dianions acting as hydrogen-bond acceptors.

In the title compound, cation, anion and uncoordinated water molecule have all crystallographically imposed twofold axis. As shown in Fig. 1, four water molecules coordinate to the cobalt(II) ion in the equatorial positions with Co—O bonds ranging from 2.059 (3) to 2.110 (2) Å, while two bpp ligands coordinate to the metal through N atoms [Co—N = 2.1772 (2) Å] in the axial positions to complete a slightly distorted octahedral coordination geometry. The dihedral angle between the two pyridyl planes in the cation is 86.14 (11)°, and the N···N separation is 10.169 (3) Å. The BPDS dianion does not coordinate to the cobalt(II) ion, but balances the charge. The dihedral angle formed by the symmetry-related benzene rings in the anion is 35.81 (12)°. Hydrogen bonds play an important role for enhancing the stability of the solid-state structure (Table 1). Two intermolecular hydrogen bonds are formed between oxygen atoms of the two coordinated water molecules with two oxygen atoms of sulfonate groups. Additional intermolecular hydrogen bond are formed between atom O3W of the uncoordinated water molecule and the sulfonate atom O1, and between the uncoordinated N atom of bpp and the coordinated O2W atom. All these intermolecular hydrogen bonds result in a two-dimensional layer structure (Fig. 2) parallel to the *ac* plane. The layers are further linked *via* C—H···O hydrogen bonds to give rise to a three-dimensional network (Fig. 3).

### Experimental

A mixture containing Co(NO_3_)_2_.6H_2_O (0.1 mmol), bpp (0.1 mmol), H_2_BPDS (0.1 mmol), NaOH (0.2 mmol) dissolved in water (15 ml) was sealed in a 25 ml Teflon lined stainless steel container and heated at 160 °C for 120 h. Orange crystals of (I) suitable for X-ray analysis were collected by filtration and washed with water and ethanol several times (yield 56%).

### Refinement

The water H atoms were located in a difference Fourier map and refined freely. All other H atoms were positioned geometrically, with C—H = 0.93 and 0.97 Å for aromatic and methylene H atoms, respectively, and constrained to ride on their parent atoms, with *Uiso*(H) = *xUeq*(C), where *x* = 1.5 for methyl H and *x* = 1.2 for all other H atoms.

### Crystal data

**Table:**

[Co(C_13_H_14_N_2_)_2_(H_2_O)_4_](C_12_H_8_O_6_S_2_)·H_2_O	*F*(000) = 1796
*M**_r_* = 857.84	*D*_x_ = 1.434 Mg m^−^^3^
Monoclinic, *C*2/*c*	Mo *K*α radiation, λ = 0.71073 Å
Hall symbol: -C 2yc	Cell parameters from 2386 reflections
*a* = 15.555 (3) Å	θ = 2.6–24.3°
*b* = 18.983 (3) Å	µ = 0.60 mm^−^^1^
*c* = 14.725 (3) Å	*T* = 293 K
β = 113.959 (3)°	Block, orange
*V* = 3973.3 (12) Å^3^	0.28 × 0.24 × 0.22 mm
*Z* = 4	

### Data collection

**Table:**

Bruker SMART APEX CCD area-detector diffractometer	3683 independent reflections
Radiation source: sealed tube	3035 reflections with *I* > 2σ(*I*)
graphite	*R*_int_ = 0.031
φ and ω scans	θ_max_ = 25.5°, θ_min_ = 1.8°
Absorption correction: multi-scan (*SADABS*; Bruker, 2000)	*h* = −17→18
*T*_min_ = 0.850, *T*_max_ = 0.879	*k* = −22→22
10176 measured reflections	*l* = −17→7

### Refinement

**Table:**

Refinement on *F*^2^	Primary atom site location: structure-invariant direct methods
Least-squares matrix: full	Secondary atom site location: difference Fourier map
*R*[*F*^2^ > 2σ(*F*^2^)] = 0.054	Hydrogen site location: inferred from neighbouring sites
*wR*(*F*^2^) = 0.133	H atoms treated by a mixture of independent and constrained refinement
*S* = 1.04	*w* = 1/[σ^2^(*F*_o_^2^) + (0.0615*P*)^2^ + 4.820*P*] where *P* = (*F*_o_^2^ + 2*F*_c_^2^)/3
3683 reflections	(Δ/σ)_max_ < 0.001
274 parameters	Δρ_max_ = 0.53 e Å^−^^3^
0 restraints	Δρ_min_ = −0.22 e Å^−^^3^

### Special details

**Table:**

Geometry. All e.s.d.'s (except the e.s.d. in the dihedral angle between two l.s. planes) are estimated using the full covariance matrix. The cell e.s.d.'s are taken into account individually in the estimation of e.s.d.'s in distances, angles and torsion angles; correlations between e.s.d.'s in cell parameters are only used when they are defined by crystal symmetry. An approximate (isotropic) treatment of cell e.s.d.'s is used for estimating e.s.d.'s involving l.s. planes.
Refinement. Refinement of *F*^2^ against ALL reflections. The weighted *R*-factor *wR* and goodness of fit *S* are based on *F*^2^, conventional *R*-factors *R* are based on *F*, with *F* set to zero for negative *F*^2^. The threshold expression of *F*^2^ > σ(*F*^2^) is used only for calculating *R*-factors(gt) *etc*. and is not relevant to the choice of reflections for refinement. *R*-factors based on *F*^2^ are statistically about twice as large as those based on *F*, and *R*- factors based on ALL data will be even larger.

### Fractional atomic coordinates and isotropic or equivalent isotropic displacement parameters (Å^2^)

**Table:**

	*x*	*y*	*z*	*U*_iso_*/*U*_eq_	
Co1	1.0000	0.10016 (3)	0.2500	0.03592 (19)	
N1	0.84781 (16)	0.10664 (12)	0.17038 (19)	0.0407 (6)	
N2	0.1777 (2)	0.16402 (18)	0.1131 (3)	0.0666 (9)	
O1	0.89574 (17)	0.10255 (13)	0.81063 (19)	0.0630 (7)	
O2	0.89650 (16)	0.17146 (13)	0.9492 (2)	0.0681 (7)	
O3	0.90540 (17)	0.04433 (14)	0.95901 (19)	0.0716 (8)	
O1W	1.0095 (2)	0.01810 (16)	0.3451 (2)	0.0617 (7)	
O2W	0.98303 (18)	0.17352 (12)	0.34914 (19)	0.0451 (5)	
O3W	0.0000	0.2072 (3)	0.7500	0.112 (2)	
S1	0.87135 (6)	0.10569 (5)	0.89562 (7)	0.0539 (3)	
C1	0.7473 (2)	0.10062 (17)	0.8458 (2)	0.0466 (8)	
C2	0.7022 (2)	0.04003 (18)	0.8521 (3)	0.0574 (9)	
H2	0.7370	0.0004	0.8826	0.069*	
C3	0.6055 (2)	0.03761 (17)	0.8134 (3)	0.0567 (9)	
H3	0.5756	−0.0041	0.8170	0.068*	
C4	0.5519 (2)	0.09623 (16)	0.7691 (2)	0.0445 (7)	
C5	0.5987 (2)	0.15679 (17)	0.7616 (3)	0.0507 (8)	
H5	0.5643	0.1966	0.7307	0.061*	
C6	0.6954 (2)	0.15851 (17)	0.7994 (3)	0.0515 (8)	
H6	0.7258	0.1993	0.7934	0.062*	
C7	0.7907 (2)	0.05321 (18)	0.1656 (3)	0.0565 (9)	
H7	0.8170	0.0115	0.1984	0.068*	
C8	0.6940 (2)	0.0570 (2)	0.1141 (3)	0.0654 (10)	
H8	0.6570	0.0183	0.1132	0.079*	
C9	0.6524 (2)	0.11739 (19)	0.0644 (2)	0.0511 (8)	
C10	0.7116 (2)	0.17249 (19)	0.0707 (3)	0.0524 (8)	
H10	0.6873	0.2149	0.0389	0.063*	
C11	0.8070 (2)	0.16508 (17)	0.1241 (2)	0.0460 (8)	
H11	0.8453	0.2036	0.1278	0.055*	
C12	0.5483 (2)	0.1237 (2)	0.0040 (3)	0.0688 (11)	
H12A	0.5263	0.0803	−0.0329	0.083*	
H12B	0.5376	0.1612	−0.0441	0.083*	
C13	0.4892 (2)	0.1383 (2)	0.0618 (3)	0.0536 (8)	
H13A	0.5115	0.1808	0.1010	0.064*	
H13B	0.4953	0.0995	0.1070	0.064*	
C14	0.3862 (2)	0.1472 (2)	−0.0086 (3)	0.0632 (10)	
H14A	0.3810	0.1894	−0.0477	0.076*	
H14B	0.3687	0.1077	−0.0543	0.076*	
C15	0.3155 (2)	0.15246 (17)	0.0364 (3)	0.0479 (8)	
C16	0.3368 (2)	0.1742 (2)	0.1314 (3)	0.0616 (10)	
H16	0.3985	0.1860	0.1727	0.074*	
C17	0.2674 (3)	0.1787 (2)	0.1659 (3)	0.0712 (11)	
H17	0.2846	0.1931	0.2313	0.085*	
C18	0.1574 (2)	0.1424 (2)	0.0216 (4)	0.0766 (12)	
H18	0.0951	0.1313	−0.0179	0.092*	
C19	0.2225 (2)	0.1352 (2)	−0.0194 (3)	0.0673 (11)	
H19	0.2039	0.1187	−0.0842	0.081*	
H3W	0.029 (5)	0.177 (3)	0.728 (5)	0.16 (3)*	
H2WB	1.017 (3)	0.1727 (17)	0.405 (3)	0.044 (10)*	
H2WA	0.928 (4)	0.172 (2)	0.351 (3)	0.107 (17)*	
H1WB	1.020 (3)	0.028 (2)	0.395 (3)	0.078 (18)*	
H1WA	0.972 (3)	−0.014 (2)	0.328 (3)	0.080 (14)*	

### Atomic displacement parameters (Å^2^)

**Table:**

	*U*^11^	*U*^22^	*U*^33^	*U*^12^	*U*^13^	*U*^23^
Co1	0.0247 (3)	0.0405 (3)	0.0428 (3)	0.000	0.0139 (2)	0.000
N1	0.0272 (12)	0.0478 (15)	0.0476 (15)	0.0026 (10)	0.0157 (12)	−0.0015 (12)
N2	0.0409 (17)	0.098 (2)	0.068 (2)	0.0096 (15)	0.0292 (17)	0.0128 (19)
O1	0.0485 (14)	0.0777 (17)	0.0678 (17)	0.0072 (12)	0.0288 (13)	−0.0025 (13)
O2	0.0400 (13)	0.0750 (17)	0.0736 (17)	0.0078 (11)	0.0070 (12)	−0.0204 (14)
O3	0.0547 (15)	0.0849 (18)	0.0633 (16)	0.0308 (13)	0.0116 (13)	0.0095 (14)
O1W	0.0676 (18)	0.0586 (17)	0.0534 (18)	−0.0231 (13)	0.0188 (15)	0.0049 (14)
O2W	0.0320 (12)	0.0611 (14)	0.0438 (14)	0.0003 (10)	0.0169 (12)	−0.0048 (11)
O3W	0.087 (4)	0.079 (3)	0.174 (6)	0.000	0.058 (4)	0.000
S1	0.0357 (4)	0.0678 (6)	0.0518 (5)	0.0141 (4)	0.0112 (4)	−0.0087 (4)
C1	0.0363 (17)	0.0576 (19)	0.0421 (17)	0.0101 (14)	0.0118 (14)	−0.0053 (15)
C2	0.049 (2)	0.053 (2)	0.068 (2)	0.0172 (16)	0.0222 (18)	0.0098 (17)
C3	0.052 (2)	0.0467 (19)	0.073 (2)	0.0044 (15)	0.0274 (19)	0.0087 (18)
C4	0.0389 (17)	0.0501 (18)	0.0433 (18)	0.0015 (14)	0.0154 (15)	−0.0009 (15)
C5	0.0373 (17)	0.0504 (18)	0.054 (2)	0.0036 (14)	0.0082 (16)	0.0064 (16)
C6	0.0368 (17)	0.0532 (19)	0.056 (2)	−0.0008 (14)	0.0104 (16)	0.0042 (16)
C7	0.0337 (17)	0.056 (2)	0.072 (2)	−0.0003 (14)	0.0130 (17)	0.0088 (18)
C8	0.0369 (18)	0.070 (2)	0.081 (3)	−0.0160 (17)	0.0149 (19)	0.000 (2)
C9	0.0286 (16)	0.079 (2)	0.0449 (18)	0.0056 (15)	0.0143 (15)	−0.0078 (17)
C10	0.0373 (17)	0.065 (2)	0.057 (2)	0.0164 (15)	0.0209 (16)	0.0097 (17)
C11	0.0336 (16)	0.0495 (18)	0.057 (2)	0.0038 (13)	0.0212 (15)	0.0021 (15)
C12	0.0317 (18)	0.117 (3)	0.056 (2)	0.0058 (19)	0.0162 (17)	−0.009 (2)
C13	0.0322 (17)	0.078 (2)	0.052 (2)	0.0041 (16)	0.0180 (16)	0.0006 (18)
C14	0.0362 (18)	0.098 (3)	0.056 (2)	0.0070 (18)	0.0194 (17)	−0.001 (2)
C15	0.0307 (16)	0.0598 (19)	0.0523 (19)	0.0049 (14)	0.0160 (15)	0.0025 (16)
C16	0.0310 (17)	0.095 (3)	0.056 (2)	−0.0018 (17)	0.0149 (16)	−0.009 (2)
C17	0.051 (2)	0.109 (3)	0.056 (2)	0.010 (2)	0.025 (2)	−0.002 (2)
C18	0.0328 (19)	0.111 (3)	0.087 (3)	−0.007 (2)	0.025 (2)	−0.004 (3)
C19	0.0372 (19)	0.100 (3)	0.063 (2)	−0.0022 (19)	0.0182 (18)	−0.016 (2)

### Geometric parameters (Å, °)

**Table:**

Co1—O1W^i^	2.059 (3)	C5—H5	0.9300
Co1—O1W	2.059 (3)	C6—H6	0.9300
Co1—O2W	2.110 (2)	C7—C8	1.385 (4)
Co1—O2W^i^	2.110 (2)	C7—H7	0.9300
Co1—N1^i^	2.177 (2)	C8—C9	1.373 (5)
Co1—N1	2.177 (2)	C8—H8	0.9300
N1—C11	1.322 (4)	C9—C10	1.371 (5)
N1—C7	1.331 (4)	C9—C12	1.503 (4)
N2—C18	1.318 (5)	C10—C11	1.376 (4)
N2—C17	1.321 (5)	C10—H10	0.9300
O1—S1	1.449 (3)	C11—H11	0.9300
O2—S1	1.443 (3)	C12—C13	1.511 (4)
O3—S1	1.451 (3)	C12—H12A	0.9700
O1W—H1WB	0.71 (4)	C12—H12B	0.9700
O1W—H1WA	0.81 (4)	C13—C14	1.524 (4)
O2W—H2WB	0.78 (4)	C13—H13A	0.9700
O2W—H2WA	0.86 (5)	C13—H13B	0.9700
O3W—H3W	0.87 (6)	C14—C15	1.501 (5)
S1—C1	1.766 (3)	C14—H14A	0.9700
C1—C2	1.370 (5)	C14—H14B	0.9700
C1—C6	1.371 (4)	C15—C16	1.364 (5)
C2—C3	1.376 (5)	C15—C19	1.382 (4)
C2—H2	0.9300	C16—C17	1.371 (5)
C3—C4	1.384 (4)	C16—H16	0.9300
C3—H3	0.9300	C17—H17	0.9300
C4—C5	1.388 (4)	C18—C19	1.381 (5)
C4—C4^ii^	1.479 (6)	C18—H18	0.9300
C5—C6	1.376 (4)	C19—H19	0.9300
			
O1W^i^—Co1—O1W	81.7 (2)	N1—C7—C8	122.8 (3)
O1W^i^—Co1—O2W	167.14 (11)	N1—C7—H7	118.6
O1W—Co1—O2W	91.35 (12)	C8—C7—H7	118.6
O1W^i^—Co1—O2W^i^	91.35 (12)	C9—C8—C7	120.5 (3)
O1W—Co1—O2W^i^	167.14 (11)	C9—C8—H8	119.8
O2W—Co1—O2W^i^	97.41 (13)	C7—C8—H8	119.8
O1W^i^—Co1—N1^i^	99.86 (11)	C10—C9—C8	116.3 (3)
O1W—Co1—N1^i^	85.08 (11)	C10—C9—C12	120.8 (3)
O2W—Co1—N1^i^	90.24 (10)	C8—C9—C12	122.9 (3)
O2W^i^—Co1—N1^i^	85.48 (10)	C9—C10—C11	120.0 (3)
O1W^i^—Co1—N1	85.08 (11)	C9—C10—H10	120.0
O1W—Co1—N1	99.86 (11)	C11—C10—H10	120.0
O2W—Co1—N1	85.48 (10)	N1—C11—C10	124.1 (3)
O2W^i^—Co1—N1	90.24 (10)	N1—C11—H11	117.9
N1^i^—Co1—N1	173.52 (13)	C10—C11—H11	117.9
C11—N1—C7	116.3 (3)	C9—C12—C13	115.9 (3)
C11—N1—Co1	120.9 (2)	C9—C12—H12A	108.3
C7—N1—Co1	122.8 (2)	C13—C12—H12A	108.3
C18—N2—C17	115.1 (3)	C9—C12—H12B	108.3
Co1—O1W—H1WB	116 (4)	C13—C12—H12B	108.3
Co1—O1W—H1WA	121 (3)	H12A—C12—H12B	107.4
H1WB—O1W—H1WA	110 (5)	C12—C13—C14	110.4 (3)
Co1—O2W—H2WB	120 (2)	C12—C13—H13A	109.6
Co1—O2W—H2WA	113 (3)	C14—C13—H13A	109.6
H2WB—O2W—H2WA	103 (4)	C12—C13—H13B	109.6
O2—S1—O1	113.57 (17)	C14—C13—H13B	109.6
O2—S1—O3	113.29 (16)	H13A—C13—H13B	108.1
O1—S1—O3	111.55 (15)	C15—C14—C13	117.6 (3)
O2—S1—C1	106.38 (14)	C15—C14—H14A	107.9
O1—S1—C1	105.30 (15)	C13—C14—H14A	107.9
O3—S1—C1	105.96 (16)	C15—C14—H14B	107.9
C2—C1—C6	119.5 (3)	C13—C14—H14B	107.9
C2—C1—S1	121.4 (2)	H14A—C14—H14B	107.2
C6—C1—S1	119.1 (3)	C16—C15—C19	116.3 (3)
C1—C2—C3	120.3 (3)	C16—C15—C14	123.8 (3)
C1—C2—H2	119.9	C19—C15—C14	119.9 (3)
C3—C2—H2	119.9	C15—C16—C17	119.9 (3)
C2—C3—C4	121.0 (3)	C15—C16—H16	120.1
C2—C3—H3	119.5	C17—C16—H16	120.1
C4—C3—H3	119.5	N2—C17—C16	124.8 (4)
C3—C4—C5	118.0 (3)	N2—C17—H17	117.6
C3—C4—C4^ii^	122.3 (2)	C16—C17—H17	117.6
C5—C4—C4^ii^	119.8 (2)	N2—C18—C19	124.4 (4)
C6—C5—C4	120.6 (3)	N2—C18—H18	117.8
C6—C5—H5	119.7	C19—C18—H18	117.8
C4—C5—H5	119.7	C18—C19—C15	119.4 (4)
C1—C6—C5	120.6 (3)	C18—C19—H19	120.3
C1—C6—H6	119.7	C15—C19—H19	120.3
C5—C6—H6	119.7		
			
O1W^i^—Co1—N1—C11	−121.1 (3)	C11—N1—C7—C8	1.1 (5)
O1W—Co1—N1—C11	158.2 (2)	Co1—N1—C7—C8	−179.3 (3)
O2W—Co1—N1—C11	67.6 (2)	N1—C7—C8—C9	0.3 (6)
O2W^i^—Co1—N1—C11	−29.8 (2)	C7—C8—C9—C10	−1.2 (5)
O1W^i^—Co1—N1—C7	59.3 (3)	C7—C8—C9—C12	177.6 (3)
O1W—Co1—N1—C7	−21.4 (3)	C8—C9—C10—C11	0.6 (5)
O2W—Co1—N1—C7	−112.0 (3)	C12—C9—C10—C11	−178.2 (3)
O2W^i^—Co1—N1—C7	150.6 (3)	C7—N1—C11—C10	−1.7 (5)
O2—S1—C1—C2	−134.6 (3)	Co1—N1—C11—C10	178.6 (2)
O1—S1—C1—C2	104.6 (3)	C9—C10—C11—N1	0.9 (5)
O3—S1—C1—C2	−13.7 (3)	C10—C9—C12—C13	−100.7 (4)
O2—S1—C1—C6	45.8 (3)	C8—C9—C12—C13	80.6 (5)
O1—S1—C1—C6	−75.0 (3)	C9—C12—C13—C14	177.0 (3)
O3—S1—C1—C6	166.7 (3)	C12—C13—C14—C15	172.0 (3)
C6—C1—C2—C3	−0.9 (5)	C13—C14—C15—C16	23.9 (6)
S1—C1—C2—C3	179.5 (3)	C13—C14—C15—C19	−156.5 (4)
C1—C2—C3—C4	−1.2 (6)	C19—C15—C16—C17	−0.8 (6)
C2—C3—C4—C5	2.3 (5)	C14—C15—C16—C17	178.8 (4)
C2—C3—C4—C4^ii^	−177.1 (4)	C18—N2—C17—C16	1.4 (6)
C3—C4—C5—C6	−1.5 (5)	C15—C16—C17—N2	−0.8 (7)
C4^ii^—C4—C5—C6	178.0 (4)	C17—N2—C18—C19	−0.3 (7)
C2—C1—C6—C5	1.7 (5)	N2—C18—C19—C15	−1.3 (7)
S1—C1—C6—C5	−178.7 (3)	C16—C15—C19—C18	1.8 (6)
C4—C5—C6—C1	−0.5 (5)	C14—C15—C19—C18	−177.8 (4)

Symmetry codes: (i) −*x*+2, *y*, −*z*+1/2; (ii) −*x*+1, *y*, −*z*+3/2.

### Hydrogen-bond geometry (Å, °)

**Table:**

*D*—H···*A*	*D*—H	H···*A*	*D*···*A*	*D*—H···*A*
O1W—H1WA···O3^iii^	0.81 (4)	2.60 (4)	3.008 (4)	113 (3)
O1W—H1WA···O1^iii^	0.81 (4)	2.01 (5)	2.812 (4)	169 (4)
O2W—H2WA···N2^iv^	0.86 (5)	1.93 (5)	2.779 (4)	167 (5)
O1W—H1WB···O3^v^	0.71 (4)	2.01 (5)	2.687 (4)	160 (5)
O2W—H2WB···O2^v^	0.78 (4)	2.01 (4)	2.795 (4)	179 (4)
O3W—H3W···O1^ii^	0.87 (6)	2.05 (6)	2.924 (4)	174 (7)
C10—H10···O2^vi^	0.93	2.56	3.360 (4)	144
C16—H16···O3W^vii^	0.93	2.54	3.311 (5)	141

Symmetry codes: (iii) *x*, −*y*, *z*−1/2; (iv) −*x*+1, *y*, −*z*+1/2; (v) −*x*+2, *y*, −*z*+3/2; (ii) −*x*+1, *y*, −*z*+3/2; (vi) −*x*+3/2, −*y*+1/2, −*z*+1; (vii) −*x*+1/2, −*y*+1/2, −*z*+1.
